# Division of labour as key driver of social evolution

**DOI:** 10.1098/rstb.2023.0261

**Published:** 2025-03-20

**Authors:** Michael Taborsky, Jennifer H. Fewell, Robert Gilles, Barbara Taborsky

**Affiliations:** ^1^ Behavioural Ecology Division, Institute of Ecology and Evolution, University of Bern, Hinterkappelen 3032, Switzerland; ^2^ Department of Collective Behavior, Max Planck Institute of Animal Behavior, Konstanz 78467, Germany; ^3^ Institute for Advanced Study (Wissenschaftskolleg zu Berlin), Berlin 14193, Germany; ^4^ School of Life Sciences, Arizona State University, Tempe, AZ 85287, USA; ^5^ Economics Department, The Queen’s University of Belfast, Belfast BT9 5EE, UK

**Keywords:** cooperation, division of labour, social evolution, task specialization, sociality

## Abstract

The social division of labour (DoL) has been renowned as a key driver of the economic success of human societies dating back to ancient philosophers such as Plato (in *The Republic*, *ca* 380 BCE), Xenophon (in *Cyropaedia*, *ca* 370 BCE) and Aristotle (in *Politics*, *ca* 350 BCE, and *Nicomachean Ethics*, *ca* 340 BCE). Over time, this concept evolved into a cornerstone of political economic thought, most prominently expressed in Smith (in *The Wealth of Nations*, 1776). In his magnum opus, Adam Smith posited that DoL has caused a greater increase in production than any other factor in human history. There is little doubt that DoL immensely increases productive output, both in humans and in other organisms, but it is less clear how it comes about, how it is organized and what the biological roots are of this human ‘turbo enhancer’. We address these questions here using results from studies of a wide range of organisms and various modelling approaches.

This article is part of the theme issue ‘Division of labour as key driver of social evolution’.

## 1. Introduction

The social insects, ants in particular, convey the impression that humans are not the only ones owing their great ecological success to a well-organized division of labour (DoL). In fact, the elements of DoL can be found across the diversity of social taxa, including microorganisms, invertebrates and vertebrates [1–4]. The 18 articles in this volume scrutinize the existence and significance of DoL from diverse angles, including ethology, ecology, evolutionary biology, anthropology and sociology. They outline our current understanding of the occurrence, similarities and differences of DoL in divergent taxa, ranging from microbes to humans, while exploring the underlying proximate and ultimate causes using theoretical and empirical approaches.

## 2. Conditions favouring DoL

Above all, we may ask which environmental and social conditions favour task specialization and DoL. Theory suggests that an important ecological factor selecting for specialism and DoL is environmental stability. When there is little conflict of interest between individuals and the group, in invariable environments specialization by group members can emerge as either task demand or task efficiency are varied, as revealed by simulations of Ito & Higginson [5]. Interestingly, their results also suggest selection for redundancy in the workforce, which might explain the widespread existence of idle workers in social insects. Even if this study did not explicitly consider group size effects, task choice and specialism may be strongly influenced also by this parameter due to demographic stochasticity, which affects small groups more strongly than large ones. Variation in labour demands requires individuals in small groups to switch more often between tasks, causing less optimal task distribution in the group and reduced responsiveness of individuals to resulting task imbalances [6].

Previous experience and learning are also relevant to task specialization and may be more important for members of small groups than for large groups [6]. This implies that costs associated with gathering experience and ltearning can be saved more easily in larger groups, which may be another reason why they are more prone to show division of labour than small groups. The importance of sizable groups with enduring membership for the emergence of DoL [7] is strongly corroborated by empirical evidence examining the distribution of DoL across taxa [8–11]. Moreover, both observational and experimental results show that DoL emerges more readily when group size is increased [12–15], which may enhance performance and energy efficiency [16,17], even if not necessarily at the individual worker level [18] (but see [19] for discussion).

Both temporal polyethism, referring to behavioural specialization lasting for some time in the life of group members, and caste differentiation, which describes lifelong specialization, are most prominent in the huge colonies of eusocial ants, bees and termites [8,20]. Nevertheless, group size effects for the diversification and role specialization of group members become apparent also across different taxa ranging from microbes to mammals. In bacterial colonies of *Streptomyces coelicolor*, for example, cells may specialize either in growth and sporulation or in the production of antibiotics. Mutations trigger this DoL between bacterial cells, and the mutation frequency increases with colony size and responds to environmental cues such as competitive pressure [21]. In cooperatively breeding Damaraland mole rats, behavioural differences between breeders and helpers increase with group size, where breeders in large groups seem to be somewhat relieved from energetically demanding digging duties [22]. Behavioural differences were shown to increase with group size also in harvester ants [23] and in clonal raider ants lacking caste differentiation, *Ooceraea biroi*, where defence behaviour can be experimentally initiated even among essentially identical individuals [24].

## 3. Levels of specialization and regulatory mechanisms

There are two principal levels of specialization leading to DoL: (i) the differentiation into reproductive and supportive functions; and, within the second level, (ii) the separation into different functions supporting the group and its members. ‘Reproductive division of labour’ describes the division of individuals specializing either in the production of offspring or in raising and protecting them. This distinction may be temporary as exemplified by cooperative breeders, or persist across a lifetime as demonstrated by eusocial insects. Termites exhibit the entire scale of temporal specializations into reproductive and supportive functions within one taxon [25]. In wood-dwelling species that obtain the required resources within their gallery system, all group members perform all tasks typically in an age-dependent sequence, starting from larval work and ending in the reproductive role either within their natal colony or after founding a new colony following dispersal. Species relying on food acquisition outside of the colony home exhibit a bifurcated development, with individuals specializing in either reproductive or supportive functions. If individuals specialize in supportive functions such as brood care and defence, they may either take over reproductive functions at a later stage in life or remain non-reproductive throughout their entire lives [25].

A question that has intrigued researchers for a long time is how the specialization underlying DoL comes about. In the tropical paper wasp *Ropalidia marginata*, for instance, physical dominance behaviour establishes DoL in newly founded colonies, whereas in somewhat aged colonies, the dominant, reproductive queen chemically regulates the production of non-reproductive workers. In such colonies, supportive functions are regulated by physical dominance among the workers themselves [26]. The evolution of the separation into these distinct roles is a matter of general interest. Why would selection favour individuals specializing in divergent roles when they represent undifferentiated morphological phenotypes? This question may be best addressed by studying non-social taxa [27]. Generally, specialization is assumed to improve task performance [8], which is effectively demonstrated by the enormous productivity of queens in some ant and termite species resembling virtual egg-laying machines, while their workers and soldiers are morphologically and behaviourally equipped to aptly fulfil their tasks [28,29]. However, this seems to correspond to an end point, the route to which is worthwhile to be envisioned. Initially, efficiency gains may be achieved by experience [30,31] and reduced metabolic and cognitive costs associated with switching between functions [19,32]. The magnitude of these efficiency gains may further depend on the time and resource investment associated with the divergent roles, and physiological limitations may constrain effective change between them [27,33].

How can these different roles be coordinated within a society so that redundancy is restrained and potential synergies are enhanced? It seems likely that cues indicating societal demand for tasks, along with the current distribution of tasks, influence individual biases toward specific task preferences, as shown for instance in bumble bees (*Bombus terrestris*) [34]. The ‘helping niche specialization hypothesis’ further proposes that this process may begin already during early ontogeny, even before helping behaviours are actively performed, and that under certain conditions it may initiate life-long task differentiation and specialization [35]. Once individuals execute tasks, their performance can be reinforced by experience, which may also reduce response thresholds to task demands [24,36–38]. Inter-individual variability in response thresholds resulting in task specialization and DoL can be influenced by genetic factors, as demonstrated in honey bees [39–42], but if group members are able to perform different tasks, experience and learning can also be important mechanisms underlying specialization [6,38–43]. In clonal raider ants (*O. biroi*), the social context is key to the emergence of DoL among genetically identical subjects [24], and in microbes, experimental results suggest that environmental demands can influence the evolution of task specialization via a change in mutation frequency or gene regulation [21].

In the early stages of sociality, DoL evolves typically ‘bottom up’, without regulatory forces exerted by group members [7,27,43,44]. This implies that task specialization is not controlled by a central agency but emerges through self-organization from the interactions of group members with their environment and among each other [37,45,46]. If the costs of switching tasks are high, evolutionarily stable task specialization and DoL may evolve [47,48]. Even among morphologically similar group members, subtle specializations may emerge, like in the nest defence behaviour of honeybees [42]. Age and size effects may be important for specialization into different tasks [35], as shown, for instance, in termites [25], ants [49], cooperatively breeding fishes [50–52] and African mole rats [22,53,54]. At later stages of the evolution of sociality, dominance, manipulation and enforcement may strongly affect task performance, specialization and DoL based on power asymmetries [55,56].

## 4. DoL within and between groups and species

Commodities are not only exchanged within social groups but also beyond, most noticeably in mutualistic interactions between different species. The involved ‘outsourcing of functions’ bears an important resemblance to within-group division of labour [57,58]. Between-species mutualisms and DoL within social groups share the common thread that different individuals contribute to the relationship through some form of specialization. In both contexts, the contributed specialization can range from short term and facultative to lifelong and obligate, and inclusive fitness benefits to both parties are expected for the relationship to persist. Social cooperation and mutualisms are often intertwined. As illustrated by the fungus-tending social insects (the fungus-tending termites, leafcutter ants and ambrosia beetles), as well as the impact on sociality by the microbiome, social organization is often interdependent with mutualism [58–64]. These relationships remind us of the general case that sociality itself is dependent on the ecological context in which it evolves.

A clear distinction between DoL within a society and material exchanges between different species is that in the latter case, agents do not belong to the same gene pool, prompting comparisons of interspecific mutualism with non-kin-based social cooperation. The success of mutualisms as between-species cooperation shows how different services and commodities can be exchanged among unrelated agents if the payoffs are somewhat correlated, similar to situations involving conspecifics [55,65,66]. Both in social cooperation and interspecific mutualisms, this does not mean that all participants are similarly rewarded in such exchanges. Trading and negotiations often involve asymmetries, potentially causing some sort of social control and enforcement [67–69], and can set the stage for manipulation, coercion and parasitism in between-species interactions as well as within social groups [58,70,71].

The question of when and how DoL can create within-group asymmetries extends also to groups of relatives, because competition between individuals of a kin group can be similarly important as among unrelated group members [72–75]. This prompts the question under which conditions subordinate individuals should be tolerated in a group and to which degree this depends on the competition for reproduction and the distribution of tasks within the group. A kin selection model of Rodrigues & Riehl [76] suggests that cooperative breeding involving delayed dispersal of non-reproducing subordinates can be selected even if helpers gain neither direct fitness via increased survival nor indirect fitness via helping the brood or their parents. The tolerance of subordinates by dominant breeders can be favoured if they are related (e.g. own offspring), irrespective of whether they take over tasks that would further enhance the breeders’ reproductive success. Nevertheless, subordinate group members should be under selection to increase the fitness of their relatives by providing help, which can consequently lead to non-reproductive DoL within groups [76].

## 5. The human case

The basic idea why task specialization and DoL may emerge is the potential enhancement of performance and productivity—exactly what Adam Smith proposed for human economies 250 years ago [77]. Nevertheless, for modern human societies, Diehl & Preisendörfer [78] cast doubt on the predominant importance of efficiency gains from increasing specialization. By pointing out inherent problems and disadvantages of specialized work, both at the group and individual levels [79–81], one should stress that in human societies, social norms, socio-economic institutions and power are similarly important drivers of organizational practices and, ultimately, DoL. In that regard, efficiency and social norm compliance can be considered complementary [82]. Indeed, like in social exchanges between animals [69], power and coercion may play an additional important role in the organization of work in human societies [83,84]. The triadic influence of efficiency considerations, social norm compliance and power asymmetries can be illustrated by the division of labour within households of contemporary couples [78,85]. In contrast, the importance of DoL in the context of organizing it through a wage-based labour market for modern, globalized economies seems to dwindle [86–89]. This is also exemplified by questions related to the value of work, or its absence, in the globalized economy [90,91].

The extensive and sometimes irreversible task specialization found in social insects is paralleled in human societies, where DoL often leads to the production of goods that cannot be recreated by naive generalists. Such cultural ratcheting [92] may generate behaviourally differentiated and strongly interdependent individuals and groups that distribute the cognitive and physical costs of production among distinct specialists, thereby resulting in irreversible specializations referred to as ‘social ratcheting’ [93]. Agent-based simulations show that limited individual memory capacities in connection with social transmission may have an important accelerating effect on the cultural evolution of material and social differentiation among producers [94]. The ability to learn from others and to use a network of agents fulfilling different functions is one important reason for the ecological success of *Homo sapiens* [95]. This virtue may extend beyond the core social unit, thereby greatly extending the capacity to gain from profitable exchanges among specialized individuals and groups [94,96], which, in fact, is not limited to humans [97,98].

## 6. Conclusions

We may ask whether and to which extent the division of labour is a key driver of social evolution, as suggested by the title of this volume. The specialization of individual group members into different tasks combined with the exchange of goods and services between them to fulfil their needs is doubtlessly a major challenge. Social complexity supersedes individual complexity in the tradeoff between individual and collective performance and cognition [20,99]. With increasing group size, DoL becomes more profitable, which involves greater organizational requirements, enhances complexity and in addition creates positive feedback on group size [17,100,101]. As early as 100 years ago, J. B. S. Haldane and Julian Huxley realized that the joining of separate units to form a colony and associated division of labour greatly enhances biological complexity by ‘turning a mere aggregation into an individual’; they hinted at coral polyps as an example [102, p. 235]. Meanwhile, it is undisputed that cooperation and a division of labour between separate units are the basis of major transitions in evolution [103], largely resulting from synergisms caused by significant efficiency advances through the combination of different functions [104]. Ultimately, these processes can lead to the formation of obligate symbioses and, in fact, multicellular organisms [58,105–108]. Individual specialization and division of labour can stabilize societies and may be seen as a one-way street toward social complexity, with retrogression getting increasingly improbable the more individual agents are connected by their mutual dependence on each other’s functions [7].

## Data Availability

This article has no additional data.
